# Sampling circulating tumor cells for clinical benefits: how frequent?

**DOI:** 10.1186/s13045-015-0174-9

**Published:** 2015-06-25

**Authors:** Sai Mun Leong, Karen ML Tan, Hui Wen Chua, Doreen Tan, Delly Fareda, Saabry Osmany, Mo-Huang Li, Steven Tucker, Evelyn SC Koay

**Affiliations:** Department of Laboratory Medicine, National University Hospital, Level 3 NUH Main Building, 5 Lower Kent Ridge Road, Singapore, 119074 Singapore; Department of Pathology, National University of Singapore, Level 3 NUH Main Building, 5 Lower Kent Ridge Road, Singapore, 119074 Singapore; Tucker Medical, Novena Specialist Center, 8 Sinaran Drive #04-03, Singapore, 307470 Singapore; Radlink PET and Cardiac Imaging Center, 290 Orchard Road, #08-06 Paragon Medical, Singapore, 238859 Singapore; CellSievo Private Limited Singapore, Block 289A, Bukit Batok St. 25, #15-218, Singapore, 650289 Singapore

**Keywords:** Circulating tumor cells, Therapeutic monitoring, Cancer, Sampling interval, Monitoring frequency

## Abstract

Circulating tumor cells (CTCs) are cells shed from tumors or metastatic sites and are a potential biomarker for cancer diagnosis, management, and prognostication. The majority of current studies use single or infrequent CTC sampling points. This strategy assumes that changes in CTC number, as well as phenotypic and molecular characteristics, are gradual with time. In reality, little is known today about the actual kinetics of CTC dissemination and phenotypic and molecular changes in the blood of cancer patients. Herein, we show, using clinical case studies and hypothetical simulation models, how sub-optimal CTC sampling may result in misleading observations with clinical consequences, by missing out on significant CTC spikes that occur in between sampling times. Initial studies using highly frequent CTC sampling are necessary to understand the dynamics of CTC dissemination and phenotypic and molecular changes in the blood of cancer patients. Such an improved understanding will enable an optimal, study-specific sampling frequency to be assigned to individual research studies and clinical trials and better inform practical clinical decisions on cancer management strategies for patient benefits.

## Circulating tumor cells

Circulating tumor cells (CTCs), shed from primary or metastatic tumor into the blood [1], may represent the source of metastases and hence allow a “liquid biopsy” for molecular characterization to further our understanding of the molecular mechanisms underlying metastasis [2]. In the past decade, advances in technology have allowed the development of assays for CTCs as biomarkers for disease progression and therapeutic response [3]. Numerous studies using different assays have shown that CTCs may be used to predict disease progression and survival in metastatic and possibly even early-stage cancer [4–16]. With the advent of precision medicine, CTCs are currently being pursued as real-time indicators for disease monitoring.

Currently, most studies on CTCs generally employ single or infrequent CTC sampling points [4–16], with the implicit assumption that changes in CTC number, as well as phenotypic and molecular characteristics, are gradual with time. In reality, little is known about the kinetics of these changes in the blood of cancer patients, and the frequencies and time points chosen for their sampling are usually arbitrary and not scientifically validated. Herein, we review examples from literature and our own case studies to show how sub-optimal CTC sampling may result in misleading observations with clinical consequences. The measurement of CTCs is currently not yet recommended in cancer guidelines for diagnosis or to influence treatment decisions [17]. In exploring CTCs as biomarkers of disease progression and therapeutic monitoring, the question of when and how often to sample needs to be answered before CTCs can be used to influence therapeutic decisions.

CTCs were first described in 1869 [1], but the clinical relevance of CTCs was only demonstrated by Cristofanilli et al. in 2004 in metastatic breast cancer patients [4]. In that study, a single measurement of >5 CTCs before treatment was shown to be an independent predictor of progression-free survival (PFS) and overall survival (OS) in metastatic breast cancer [4]. More recently, studies in patients with non-metastatic breast cancer also demonstrated that the presence of ≥1 CTC predicted early recurrence and decreased OS [5]. The prognostic value of CTCs was also investigated for other cancer types such as prostate, colorectal, lung, and ovarian cancers [6–9], where CTC numbers at a single time point and exceeding a defined cutoff value predicted for increased mortality.

Subsequent studies enumerating CTCs using different assays, at multiple time points before, during, and after treatment in breast, prostate, and colorectal cancers showed that elevated CTC levels measured at any time during the treatment course predicted for disease progression [12–15]. Conversely, reduction in CTCs post-treatment was associated with improved PFS and OS [14–16], suggesting that CTCs represent an indicator of treatment failure and disease progression.

## “Saw-toothed” response

Apart from enhancing prognostication, the use of multiple time points for sampling CTCs may reveal insights into the nature of CTC dissemination in patients’ blood. In 2008, using multiple time point measurements, Pachmann et al. described three typical patterns of changes in CTCs observed for non-metastatic breast cancer patients undergoing adjuvant treatment: *pattern 1*—decrease in circulating epithelial tumor cells (CETC) count (>10-fold), *pattern 2*—marginal changes in count (<10-fold), and *pattern 3—*an increase or initial decrease with subsequent increase (“saw-toothed” pattern) (>10-fold) [18]. Notably, the pattern of change in CETC count during therapy was highly predictive of outcomes, with a highly significant increased relapse-free survival rate for patients with pattern 1 compared to pattern 2, and for patients with pattern 2 compared to pattern 3 [18]. The stratification of patients by their patterns of CTC changes by Pachmann et al. contrasts greatly with most previous studies that used defined cut-off values [4–9] and suggests that the pattern of CTC change itself may be an important predictor for patient outcomes.

Similar saw-toothed patterns were reported in subsequent studies employing different CTC isolation strategies, including the clinically validated FDA-approved CellSearch assay [19–22]. Taken together, these data appear to indicate that saw-toothed-like CTC count patterns may be a common phenomenon occurring in cancer patients undergoing therapy. The mechanism behind this phenomenon is currently unknown, but may be related to differential drug sensitivities of the different intra-tumoral regions that occur as a result of clonal heterogeneity within the primary tumor [23].

The saw-toothed count pattern suggests that CTC dissemination in the blood is not always continuous but may occur in spurts during therapy. It thus follows that depending on the time of CTC measurement, different CTC counts will be obtained and in turn, different conclusions will be drawn with regard to the nature of the patient’s real-time therapeutic response. This may explain why some studies, employing very few time points of CTC measurement during therapy, appear not to support a correlation between CTC response and tumor response. For example, Pierga et al. [24] examined the changes in CTC count before and after neoadjuvant therapy for stages II and III breast cancer patients and showed that changes in CTC count did not correlate to complete pathologic response, although CTC detection was proven to be an independent prognostic factor for early relapse [24]. Similar results were reported by Riethdorf et al. [25] for the GeparQuattro trial, which likewise employed a single measurement each for pre- and post-neoadjuvant therapy [25]. In the landmark study by Cristofanilli et al., only 50 % of patients with progressive disease had increased CTC counts [4]. It cannot be ruled out that transient rises in CTC counts occurred in the other 50 % of the patients, but were missed as only a single time point was used in the study. Uncertainty regarding the timing of CTC measurements might thus limit the predictive capacity of single or few time point CTC measurements.

## The issue of optimal sampling frequency

**Fig. 1:**
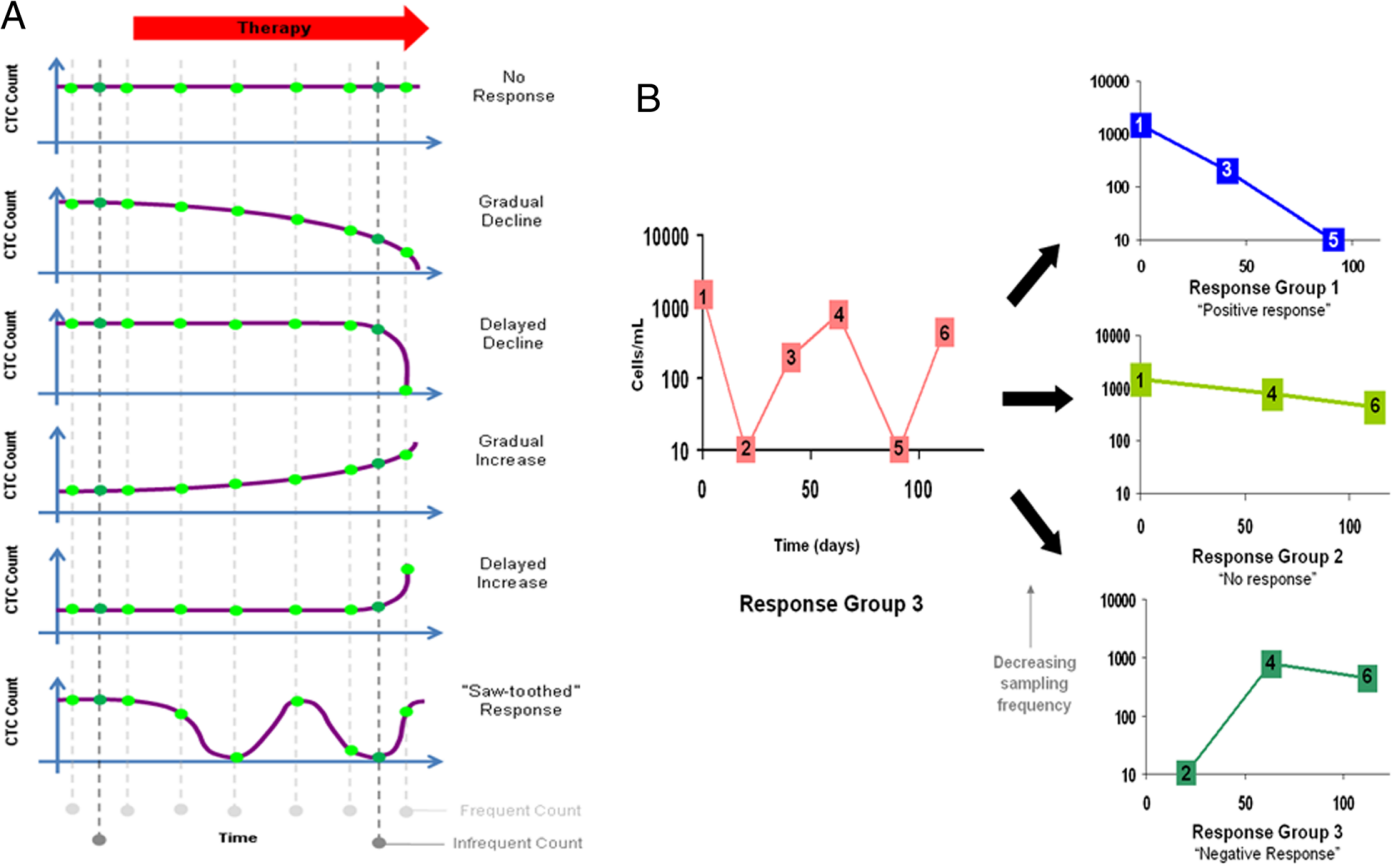
CTC response patterns in cancer patients undergoing therapy. **a** Hypothetical CTC count patterns corresponding to patient response to therapy. The *light green circles* represent frequent sampling times, and the *dark green circles* represent infrequent sampling times. **b** CTC count pattern of a breast cancer patient from response group 3 of Pachmann et al. [18]. CTC count patterns are simulated when the frequency of sampling is halved from six to three sampling points. The sampling points chosen for each simulation are numbered

**Fig. 2:**
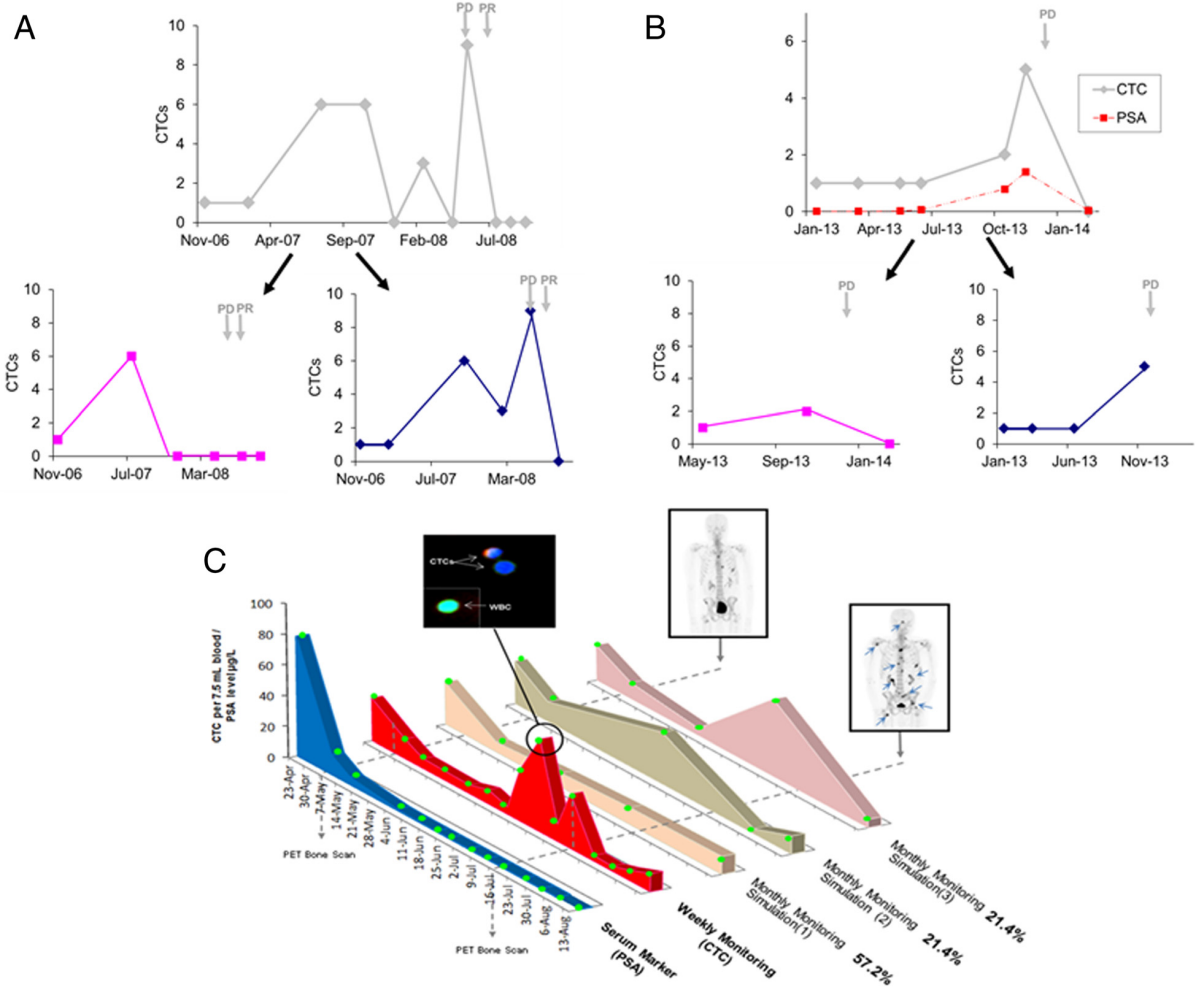
Case studies of CTC count patterns in cancer patients. **a** CTC count pattern of a metastatic breast cancer patient from Marsland and Schuur [19]. CTC count patterns are simulated when the frequency of sampling is halved. Alternate time points were used for each simulation. *PD* indicates progressive disease and *PR* indicates partial response. **b** Longitudinal monitoring of CTC count and PSA level in a prostate cancer patient. CTCs were measured using the CellSearch assay. CTC count patterns are simulated when the frequency of sampling is halved. Alternate time points were used for each simulation. *PD* indicates progressive disease. **c** Longitudinal monitoring of CTC count and PSA level in a metastatic prostate cancer patient. CTCs were imaged at ×20 magnification and identified as Hoechst (*blue*) positive and CD45 (*green*) negative, and white blood cells (WBCs) as both Hoechst and CD45 positive. Some CTCs were also EpCAM/cytokeratin (*red*) positive, while WBCs were EpCAM/cytokeratin negative. The foremost graph shows concentration of PSA with time. The second graph behind shows the CTC count over time. The third to fifth graphs behind are representatives of simulated models for monthly CTC counting. The *green circles* represent sampling times. The proportion of simulations represented by each model is indicated as percentage. A [^18^F]NaF-PET bone scan in July 2012 vs. April 2012 showed progression of osseous disease including new lesions (as indicated by *blue arrows*)
- (iii) We monitored our own prostate cancer patient using the CellSearch assay. A spike in CTCs from 1 to 5 was observed within 1 month, which correlated with prostate-specific antigen (PSA) levels and disease progression by PET/CT imaging (Fig. 2b). We simulated the count patterns observed when the frequency of blood sampling is halved, using alternate time points. The two different count patterns produced show that the spike in CTCs could be missed if CTC sampling were less frequent (Fig. 2b).
- (iv) Another case is our own patient with metastatic prostate cancer, who had weekly CTC sampling at each chemotherapy session. CTCs were isolated using a previously published size-exclusion method [26]. Using this method, which is not dependent on epithelial markers, mesenchymal CTCs that have been associated with therapeutic resistance and disease progression [21] can be captured together with the epithelial ones. For this patient, transient saw-toothed-like spikes in CTC count, with a duration span of not more than 1 month, were observed, and these transient increases preceded progression of osseous disease as shown by a PET bone scan (Fig. 2c). We performed a simulation test to determine the CTC count pattern generated when the sampling frequency was hypothetically decreased from weekly to monthly. Different start dates and intervals of 4–7 weeks between CTC measurements were simulated. Interestingly, 57.2 % (8 out of 14) of the simulated models generated failed to recapitulate the saw-toothed pattern of response group 3. In fact, these simulated count patterns (“Simulation 1”, Fig. 2c) resemble more closely the invariable pattern of response group 2 in Pachmann et al.’s study [18].

Given that different CTC count patterns may predict for significantly varied relapse-free survival [18], it will be important to distinguish between patients with invariable, gradual, or rapid decline, gradual or rapid increase, or saw-toothed patterns of CTC response. As shown in Fig. 1a, these different patterns cannot be distinguished by infrequent counts. The effect of sub-optimal count frequency on the patient’s CTC response pattern is further illustrated by the following case studies:

- (i) Using a typical saw-toothed response curve of a breast cancer patient from response group 3, as presented by Pachmann et al. [18], we simulated the count patterns observed when the frequency of blood sampling is halved. Depending on the sampling dates chosen, the patient could have a pattern resembling that of response group 1 (decrease in cell numbers), response group 2 (consistent cell numbers), or response group 3 (increase in cell numbers) when the number of sampling points is decreased from six to three points (Fig. 1b).
- (ii) A recent case report of a metastatic breast cancer patient by Marsland and Schuur [19] demonstrated a saw-toothed pattern of CTC counts during the patient’s course of therapy (Fig. 2a). CTCs were sampled three-monthly in 2007 and monthly in 2008. Using the CellSearch assay, a spike of CTCs from 0 to 8 was observed in May 2008, which corresponded with disease progression (Fig. 2a). We simulated the count patterns observed when the frequency of blood sampling is halved, using alternate time points. Depending on the sampling dates chosen, the patient could appear to have no CTCs during disease progression in 2008 (Fig. 2a).

All four case studies, using different CTC isolation technologies including the FDA-approved CellSearch assay, demonstrate how patients using a sub-optimal count frequency may end up with different count patterns indicative of different therapeutic responses and varied predictions of relapse-free survival. Depending on the start date and sampling points chosen, there is a good chance that the physician may be misled into concluding that the patient is responding to treatment, when the latter may in fact be developing resistance to therapy. Published studies typically measured CTCs at monthly intervals during therapy to assess the patients’ responses [19–21]. However, as there is still a lack of understanding of the kinetics of CTC dissemination and turnover in blood in response to treatment, it is unknown if a monthly frequency offers sufficient analytical resolution in deciphering the patient’s therapeutic response pattern based on changes in CTC number.

## CTC enumeration is only the tip of the iceberg

The above case studies demonstrate the possibility of misleading interpretation of disease progression and treatment responses based on CTC numbers, given insufficient sampling. This assumption is made purely based on CTC total numbers. Apart from the choice of sampling frequency, other factors may further confound attempts to decipher the patients’ actual therapeutic responses based on CTC number. One such factor is the technology used in isolating CTCs from patients’ peripheral blood samples. Currently, the understanding of CTCs is very much dependent on the technological approaches used for their detection and isolation. Many different CTC detection technologies have been developed, including nucleic acid-based detection [27, 28], detection based on physical properties such as larger size of epithelial cells [29], differences in density [30], charge [31], migratory properties [32, 33], and properties of specific cell types [34, 35]. Approaches of isolation of CTCs by virtue of their increased size using filtration are limited by the variability in heterogeneous CTC sizes. The most widely used CTC isolation approach relies on antibody-based capture of CTCs which express epithelial cell surface markers such as the epithelial cell adhesion molecule (EpCAM) that are absent from normal leukocytes [4, 32, 33]. The FDA-approved CellSearch system (Veridex) which uses this approach is the most standardized platform but suffers from low sensitivity [36]. This approach is also limited by the failure to detect CTCs that have undergone epithelial-mesenchymal transition (EMT), a key process involved in metastasis [37].

Given the lack of a gold standard to compare CTC isolation technologies to, it remains a question whether current methods are detecting all CTCs reliably [38]. Even though technological advances have improved CTC isolation efficiency, due to the universal issue of leukocyte contamination, identification of all CTCs with heterogeneous expression markers can be challenging. Furthermore, not all CTCs detected may be clinically relevant [38]. Patients with benign inflammatory conditions may have viable circulating epithelial cells detected by current CTC assays [39]. Because some CTCs may be undetected and some detected CTCs may not be clinically relevant, CTC enumeration by itself may not be a good marker for disease progression. An ambiguous choice of sampling time points only serves to further undermine the utility of CTC counting for clinical use. To this end, further characterization of the detected CTCs by size, the presence of clusters [40], or other phenotypic or molecular properties may add information for reliable prediction of treatment responses.

## Sampling for molecular characterization

CTCs may be shed from different sites within the tumor, which are heterogeneous, or from metastases. Molecular characterization of the CTCs such as expression profiling to detect organ-specific metastatic signatures may aid diagnostic and therapeutic strategies [38]. Molecular analysis of CTCs may provide a “real time” noninvasive approach for tumor cell genotyping, which can be repeated during the course of therapy to monitor the acquisition of novel genetic abnormalities in response to drug exposure. Moreover, different CTC parameters can influence how CTCs reflect disease progression. The proportion of cells in proliferative or apoptotic status varies during treatment and across patients [41]. The androgen receptor (AR) subcellular localization varies during treatment of androgen deprivation therapy [42]. A simple enumeration of CTCs without molecular and/or phenotypic characterization may lead to wrongful clinical assumptions and consequences. Like CTC enumeration, appropriate choice of sampling frequency for molecular and/or phenotypic characterization may be necessary for accurate interpretation of the patients’ responses to therapies. In view of this, we describe below two hypothetical examples of how insufficient sampling may demonstrate the possibility of misleading interpretation of disease progression and treatment responses based on molecular characterization of CTCs.

**Fig. 3:**
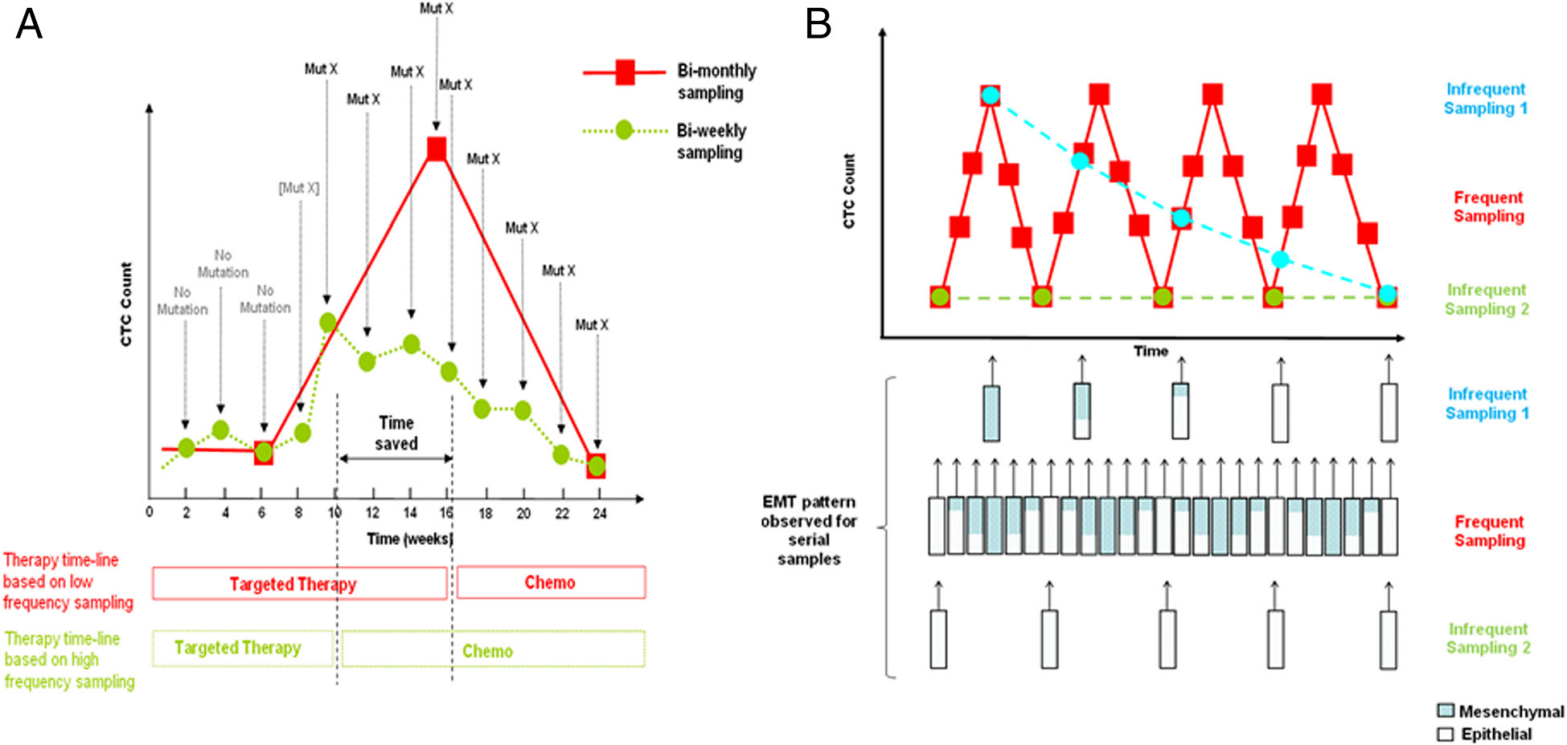
Hypothetical models of different sampling times for molecular assays of CTCs. **a** CTC count pattern and resistance mutation (Mut X) detection in a hypothetical cancer patient on targeted therapy. Mut X indicates presence of the mutation at low allele frequency. CTC count patterns and resistance mutation detection are simulated when the frequency of sampling is increased from once in 2 months (*red squares*) to once in two weeks (*green circles*). The *bars* below show the impact of earlier resistance mutation detection on choice of therapy. The *red bars* indicate the therapeutic strategy with infrequent CTC sampling while *green bars* indicate the therapeutic strategy with frequent CTC sampling. Time saved will vary, depending on when Mut X is detected at high allele frequency between week 6 and 15. **b** CTC count pattern and epithelial-mesenchymal phenotypes in a hypothetical patient with CTCs oscillating between the epithelial and mesenchymal phenotypes. Frequent sampling time is indicated in *red*, while infrequent sampling times are simulated in *blue* (infrequent sampling 1) and *green* (infrequent sampling 2). Infrequent sampling 1 and 2 differ in their start time for first sampling. The proportion of mesenchymal phenotype is indicated in the *bars below in blue* while that of epithelial phenotype is indicated in *white*

Advanced CTC analysis, such as genomic profiling, will likely increase the clinical value of CTCs as biomarkers and therapeutic targets. The use of targeted therapies has achieved considerable success; however, many patients relapse due to drug resistance [43]. The detection of small numbers of resistant CTCs could prompt the clinician to use alternative treatment strategies that might prevent resistant clones from expanding to dominate the tumor cell population [43]. Using serial CTC samples from lung cancer patients, Maheswaran et al. demonstrated evolution of *T790M* and other epidermal growth factor receptor (EGFR)-activating mutations during treatment with the tyrosine kinase inhibitor, Gefitinib [20]. The *T790M* missense mutation occurs within the EGFR kinase domain and prevents binding of Gefitinib to this region while preserving catalytic activity [44]. In their study, the sampling interval used varied from 30 to 100 days, which corresponds to 2–7 bi-weekly cycles of chemotherapy [20]. To save the patient a few cycles of ineffective therapy, we propose increasing the frequency of CTC sampling, which may permit earlier detection of *T790M* emergence. This, in turn, will enable earlier initiation of subsequent chemotherapy to eradicate the resistant clones, which may improve survival outcome [45, 46]. In a hypothetical situation as depicted in Fig. 3a, the choice of a bi-weekly monitoring interval over a bi-monthly one saves the patient about 6 weeks of ineffective treatment with the targeted therapy, due to earlier detection of the mutation conferring resistance with more frequent sampling. Subsequent therapy can then be initiated earlier, and earlier time to chemotherapy initiation of 4–8 weeks has been shown to confer significant overall survival benefit in patients with colorectal and breast cancers [47–49].

Besides mutation analysis, other molecular assays of CTCs have also been investigated as indicators of treatment response. Using an RNA in situ hybridization assay on CTCs isolated from an index breast cancer patient, Yu et al. demonstrated association of disease progression and treatment failure with EMT [21]. In this study, CTCs were assayed at 1–3-monthly intervals, with the mesenchymal phenotype correlating positively with CTC numbers [21]. Similar to CTC count, the dynamics of EMT in relation to metastasis and chemotherapeutic treatment is not well understood. As shown in a hypothetical situation (Fig. 3b), for a patient with CTCs oscillating between epithelial and mesenchymal phenotype with time, inadequate sampling may give rise to a misleading trend, e.g., an apparent mesenchymal-to-epithelial phenotypic change of “infrequent sampling 1” can give a false impression of therapeutic responsiveness, while a perpetual epithelial phenotype with low CTC count of “infrequent sampling 2” can mislead physicians into thinking that the disease is under remission.

## Perspective

The case studies presented above highlight the importance of understanding the precise kinetics of CTC dissemination before optimal count frequency can be assigned for large-scale trials and future clinical practice. As shown in the two hypothetical examples above, without appropriate frequency of CTC sampling, molecular analysis of CTCs may lead to potentially wrong assumptions or delayed diagnosis with clinical consequences. To this end, we propose that initial systematic studies involving highly frequent (e.g., weekly) sampling to understand the kinetics of CTC dissemination in patients of different cancer types, stages of disease, and treatments. A high-resolution “count-scape” will permit visualization of the actual CTC response patterns manifested in different scenarios. In the case of patients with saw-toothed responses, measurement of the range of duration spans of the CTC spikes may then lead to better understanding of the kinetics of CTC spurts and enable determination of the optimal sampling intervals to employ. This systematic approach is perceivably superior to the arbitrary manner in which sampling frequencies are currently being selected and will likely lead to a better gauge of the actual therapeutic responses based on changes in CTC count. While the high cost and resource requirements associated with highly frequent CTC sampling may be prohibitive, these may be overcome in the near future by technological advances leading to low-cost and high-throughput CTC isolation devices. Before CTC-based molecular markers can be translated to the real-world clinical setting, systematic studies with high-frequency sampling of CTCs will likely be required to establish the dynamics of the phenotypic and genetic changes occurring in CTCs.

CTCs capture real-time information about the biology of the cancer; changes in CTCs can thus reveal changes in disease status. The ultimate goal in clinical application of CTCs is its incorporation into disease management strategies. CTCs may be used, in conjunction with radiologic imaging, serum tumor markers, and clinical assessment, for real-time monitoring of disease status and therapeutic efficacy. At the moment, the sampling frequency represents a major conundrum and an unresolved unknown in the study and clinical application of CTCs. Current studies in CTC are probably limited in sampling frequency more as a result of cost and resource implications than scientific rationale. To fulfill the full clinical potential of CTCs, an initial study involving high-frequency blood sampling appears to be the unavoidable trip that all CTC researchers must take to ensure there is no loss of crucial information during time-course investigations.

## Ethics committee approval

Blood was obtained from patient described in case study (2) with approval from the Institutional Review Board (IRB) of National University of Singapore (NUS), following written informed consent.

## Abbreviations

CT: computed tomography
CTC: circulating tumor cell
CETC: circulating epithelial tumor cell
EGFR: epidermal growth factor receptor
EpCAM: epithelial cell adhesion molecule
EMT: epithelial-mesenchymal transition
PR: partial response
PFS: progression-free survival
PD: progressive disease
OS: overall survival
PET: positron emission tomography
PSA: prostate-specific antigen
WBC: white blood cell
